# Three-Dimensional Analysis of Volumetric Changes During Palatal Wound Healing After Soft-Tissue Harvesting

**DOI:** 10.3390/s26165088

**Published:** 2026-08-11

**Authors:** Viviana Desantis, Marco Brando Mario Paracchini, Fulvio Gatti, Carlo Pezzoli, Elena Maria Varoni, Marco Marcon

**Affiliations:** 1 Dipartimento di Scienze Biomediche, Chirurgiche ed Odontoiatriche, Università degli Studi di Milano, 20122 Milano, Italy; viviana.desantis@polimi.it (V.D.); fulvio.gatti@unimi.it (F.G.); 2 Dipartimento di Elettronica, Informazione e Bioingegneria, Politecnico di Milano, 20133 Milano, Italy; marcobrando.paracchini@polimi.it (M.B.M.P.); carlo.pezzoli@polimi.it (C.P.)

**Keywords:** intraoral optical sensing, intraoral scanner, 3D surface sensing, structured-light scanning, wound healing monitoring, palatal donor site, soft-tissue volumetric analysis, computer vision, digital dentistry

## Abstract

The growing demand for aesthetic and functional outcomes in periodontal and implant procedures has increased the use of connective tissue grafts harvested from the palate. Objective monitoring of the donor site remains challenging because healing involves progressive three-dimensional morphological changes and visible chromatic variations of the mucosal surface. This pilot feasibility study proposes an optical sensing and computer-vision workflow based on longitudinal intraoral scanner acquisitions to quantify palatal wound healing after soft-tissue harvesting. A TRIOS 3 intraoral scanner was used as a non-invasive sensing device to acquire textured three-dimensional datasets of the palatal donor area at baseline, immediately after surgery, and during follow-up at 1 and 2 weeks and at 1 and 3 months. The scanner output, consisting of surface geometry and color information, was processed as multimodal 3D data. The workflow included mesh preprocessing, reference-based registration of serial scans, robust alignment using geometric, normal, and chromatic cues, region-of-interest identification, and signed volumetric difference computation. Sensor-related uncertainty was addressed through an additional in vivo repeatability assessment on healthy volunteers and by comparison with previously published accuracy and precision data for the TRIOS scanner series. The resulting volumetric maps and healing curves enabled objective quantification of local tissue loss, swelling, and progressive recovery over time. Chromatic information from the textured scans was also considered as an additional sensing descriptor of mucosal healing. By integrating intraoral optical sensing, three-dimensional surface reconstruction, multimodal mesh registration, and quantitative volumetric analysis, the proposed approach provides a preliminary non-invasive framework for monitoring palatal donor-site healing. Six patients undergoing palatal soft-tissue harvesting were monitored using a TRIOS 3 intraoral scanner at baseline, immediately after surgery, and during follow-up at 1 and 2 weeks and at 1 and 3 months. Although all clinically evaluated patients exhibited complete clinical re-epithelialization by 3 months, quantitative 3D analysis revealed a persistent residual volumetric deficit, indicating that visual healing does not necessarily correspond to complete soft-tissue volume restoration. This sensor-based methodology may support clinical follow-up, improve the objectivity of soft-tissue assessment, and contribute to the development of digital biomarkers for oral wound healing.

## 1. Introduction

Gingival recession is increasingly observed in the general population. It is defined as the apical displacement of the gingival margin, which results in exposure of the root surface. Autogenous free gingival grafting (FGG) is one of the most widely used surgical procedures in modern dentistry for the treatment of gingival recession in areas with insufficient attached gingiva and for increasing keratinized tissue around teeth or implants [1]. The same FGG can also be used as a connective tissue graft (CTG), after de-epithelialization, in several mucogingival procedures, such as coronally advanced flap (CAF) surgery [2,3,4] and to increase the mucosal volume around dental implants [5]. However, a major drawback of this procedure is patient pain and discomfort at the palatal donor site, which heals by secondary intention over approximately 2–4 postoperative weeks. One particularly relevant aspect of this procedure is the volumetric assessment of soft tissues, especially in the graft harvesting area. This is clinically important because a second connective tissue graft may need to be harvested from the same donor site. Over the years, different techniques have been used to assess palatal soft-tissue thickness, including periodontal probes [6] endodontic files [7], anesthesia needles [8] and more invasive imaging tools as cone-beam computed tomography (CBCT) [9].

Here, we present an objective assessment technique for evaluating regeneration and healing of the palatal donor site based on quantitative analysis of intraoral scanner data. The increasing availability of these devices enables rapid and precise three-dimensional reconstruction of the oral cavity with sub-millimeter accuracy [10]. Detailed 3D reconstructions can be obtained at multiple healing stages for both the gingival mucosa and the palatal donor area. In this study, intraoral 3D scanners were used to perform a three-dimensional analysis of the volumetric changes occurring at the palatal donor site after connective tissue graft harvesting. Specifically, the aim was to analyze palatal soft-tissue regrowth in different patients during the postoperative recovery period. As a first step, the proposed technique enables automatic registration of point clouds and three-dimensional meshes of the entire oral cavity, or of a specific region of interest, acquired at different time points during healing. In the second step, it enables accurate evaluation of areas showing significant volumetric changes beyond instrumental acquisition noise and natural daily variations in mucosal morphology. These changes are related to tissue harvesting and may indicate either missing tissue involved in regeneration or transient swelling, such as edema caused by increased interstitial fluid. Correspondences between different regions in serial acquisitions are established using statistical descriptors, thereby allowing subsequent analysis of geometric variations in the affected areas throughout patient recovery. Given the limited sample size, this work should be regarded as a pilot feasibility study aimed at evaluating the applicability of the proposed three-dimensional workflow for longitudinal assessment of palatal donor-site healing.

### Related Work and Positioning

In recent years, intraoral scanners (IOSs) have rapidly expanded beyond prosthodontics and orthodontics, with increasing use in periodontal and peri-implant research to quantify soft-tissue changes over time. Recent reviews indicate that current IOSs are sufficiently accurate for monitoring soft-tissue variations. Nevertheless, scanning mobile and deformable tissues remains challenging and requires robust superimposition strategies and validation [11].

Digital monitoring of the palatal donor site after FGG or CTG harvesting has recently been addressed using three-dimensional volumetric approaches; in particular, a clinical study of palatal donor sites acquired serial intraoral digital scans up to 12 months and reported that volumetric changes were most pronounced during the early healing phase and attenuated thereafter [3]. A short-term pilot study also investigated hard palate healing after FGG harvesting using digital imaging and 3D analysis over a three-month period [12].

From a metrology standpoint, the accuracy of volumetric measurements from IOS has been studied with experimental models and physical references (e.g., gravimetric methods), emphasizing the need to separately report trueness and precision, as well as to analyze the impact of superimposition and scan strategy [13].

In Table 1, we reported some of the most significant articles published over the last decade on these activities.

Moreover, practical protocols have been proposed to compare volumetric changes between two IOS scans (including cross-manufacturer datasets), leveraging segmentation and reference-based best-fit superimposition to minimize alignment distortions [14].

In this work, we build on these clinical and methodological advances by proposing a robust registration-and-segmentation pipeline tailored to partially elastic palatal soft tissues, enabling longitudinal quantification of volumetric recovery while explicitly leveraging geometric and chromatic information from textured IOS meshes.

**Table 1:** Representative literature on digital/3D assessment of palatal donor site and soft-tissue changes (last 10 years).

Reference	Year	Data/Setting	Key Contribution
[3]	2022	Clinical, palatal donor site (serial scans up to 12 months)	Volumetric donor-site changes concentrated in early healing; association with graft dimensions.
[12]	2022	Clinical pilot, hard palate after FGG (3 months)	Short-term 3D digital analysis of palatal healing dynamics.
[15]	2024	Clinical pilot, donor + recipient (up to 6 months)	Digital technique with repeatability/reproducibility assessment (Gage R&R).
[14]	2025	Method/protocol paper	Cross-IOS volumetric comparison protocol with segmentation to reduce alignment distortions.
[16]	2024	Clinical precision study (palate vs. dentition)	Quantifies repeatability and intermediate precision of palatal scans; supports test–retest design.
[17]	2022	CBCT + surface models	3D palatal soft-tissue volume for presurgical planning; ROI-based analysis.
[13]	2019	Experimental models + gravimetric reference	Trueness/precision of volumetric IOS measurements with physical reference.

## 2. Materials

### 2.1. Intraoral Scanners (IOS)

Traditional diagnostic methods, such as visual inspection and conventional impressions, may be affected by inaccuracies, patient discomfort, and time delays. By contrast, intraoral 3D scanning provides high-resolution, real-time, non-invasive imaging that can improve the identification of abnormalities, including caries, periodontal disease, and oral cancer. This technology allows early detection of subtle morphological changes in soft and hard tissues, thereby improving treatment planning and clinical outcomes. In addition, several automated machine-learning techniques are emerging for the diagnosis of oral pathologies based on scans obtained with these devices. This development also creates opportunities for remote diagnosis in locations that are difficult to access, potentially with scans acquired by non-specialized personnel [18]. Additionally, 3D data can be seamlessly integrated with computer-aided design and manufacturing (CAD/CAM) systems, facilitating the development of personalized dental appliances and prostheses. The ability to track disease progression over time through repeatable, precise scans is another critical advantage, allowing for more dynamic and responsive patient care.

The accuracy, reliability, and ease of use of such sensors have gradually increased since their introduction to the market in the early 2000s. In particular, the introduction of iTero^TM^ and 3Shape products in 2007 led to a significant spread of these devices and their recognition as valuable tools for diagnosing oral pathologies. The ability to capture accurate 3D scans of the oral cavity with a precision of hundredths of a millimeter, and to associate the acquired meshes with detailed textures, also allows for the collection of detailed color information of the reconstructed surfaces, which can be used for various purposes in automated diagnoses through Machine Learning [19].

IOS devices, such as the 3Shape Trios 3 adopted in this study, operate by capturing detailed three-dimensional (3D) images of the intraoral environment through optical scanning technologies. These devices are equipped with advanced sensors and imaging systems that utilize structured light or laser to project a pattern onto the surface of the teeth and gums. The reflected light is captured by high-resolution cameras, allowing the scanner to record the surface geometry. The key principle is triangulation: the light source projects a pattern, and the cameras capture the deformed projection from different angles. From this data, the software reconstructs a precise 3D mesh of the intraoral structures, including both hard (teeth) and soft (gums) tissues.

The process begins with the scanner moving across the dental arch, continuously capturing data in real-time. Modern IOS rely on sophisticated algorithms to stitch together individual frames into a cohesive and highly accurate 3D model. As the scanning head moves, the software processes the data, aligning it with previous scans and eliminating any redundant or misaligned points [20,21]. This continuous data acquisition and processing minimize the need for rescan, providing an efficient workflow. All acquisitions were performed by the same experienced clinician in order to minimize operator-dependent variability. During each acquisition, particular attention was paid to obtaining complete coverage of the palatal donor site together with the adjacent teeth and stable palatal structures, which were subsequently used as anatomical references for scan registration. Before scanning, saliva was gently removed from the palatal region when necessary to improve optical acquisition. Care was taken to avoid excessive pressure of the scanner tip on the palatal mucosa, thereby minimizing soft-tissue deformation during image acquisition. The same acquisition procedure was adopted at every follow-up visit to maximize the consistency of the longitudinal analysis. All intraoral scans were acquired under standardized environmental conditions and lighting, with patients positioned in a standardized manner. Before each acquisition session, the intraoral scanner was calibrated according to the manufacturer’s instructions.

### 2.2. Accuracy and Repeatability Considerations

To support the clinical interpretability of small longitudinal volumetric differences, the expected measurement uncertainty of the acquisition and registration workflow was evaluated under clinically realistic intraoral conditions.

An additional in vivo repeatability assessment was performed on healthy adult volunteers recruited among hospital staff. Five intraoral acquisitions were obtained, and each acquisition was repeated three consecutive times using the same TRIOS 3 scanner and the same acquisition protocol adopted for the clinical study. All scans were acquired in normal acquisition mode, rather than high-resolution mode, in order to reduce acquisition time and improve feasibility and patient comfort during intraoral procedures.

The repeated scans were aligned using the same registration pipeline applied to the patient dataset. Residual point-to-surface deviations were evaluated in anatomically stable regions, mainly enamel surfaces and non-operated palatal areas. These regions were selected because they were not expected to undergo relevant morphological changes between consecutive acquisitions and therefore provided a practical estimate of acquisition and registration repeatability.

The resulting repeatability values were compared with previously published accuracy and precision data for the TRIOS scanner series. In particular, Amornvit et al. [22] evaluated TRIOS 3 in both normal and high-resolution acquisition modes using repeated scans and reported favorable trueness and precision for the TRIOS series compared with other intraoral scanners. Therefore, the published scanner-related accuracy values were used as a reference estimate for instrumental uncertainty, whereas the additional repeated in vivo acquisitions were used to verify the repeatability of the complete acquisition and registration workflow under real intraoral conditions.

It should be noted that the uncertainty reported in this study refers to linear surface deviations expressed in millimeters. It should not be interpreted as a direct volumetric error expressed in mm^3^, because volumetric uncertainty also depends on the size and geometry of the selected region of interest, local surface curvature, residual registration errors, and the connected-component selection step. Consequently, small residual volumetric differences observed at later follow-up time points should be interpreted cautiously and in relation to the expected sub-millimetric linear uncertainty of the scanner and registration workflow.

### 2.3. Mesh Alignment and Volumetric Analysis

Mesh alignment plays a fundamental role in the accurate assessment of volumetric changes, particularly when analyzing intersecting volumes in applications such as biomedical imaging, engineering simulations, and additive manufacturing. Ensuring precise alignment is essential to distinguish true morphological changes from artifacts introduced by mis-registration errors, which can significantly affect the reliability of volume assessments. Various alignment techniques, including iterative closest point (ICP) [23,24], feature-based matching [25], and landmark-based registration [26], can be employed to optimize mesh correspondence and improve measurement accuracy. The choice of alignment method depends on factors such as the complexity of the geometry, the presence of distinct features, and the quality of the initial scan data. In cases where anatomical or mechanical structures undergo changes over time, misalignment can lead to incorrect interpretations of volume differences, making robust registration techniques critical for reliable analysis.

Once the meshes are properly aligned, their intersecting regions can be analyzed to quantify differences, highlighting areas of material loss or gain. These intersections are critical in understanding structural modifications over time, such as bone regeneration in medical research, deformation in mechanical components, or wear analysis in industrial applications. In biomedical contexts, for example, aligning preoperative and postoperative scans allows researchers to assess tissue remodeling, implant integration, or the progression of degenerative conditions [27]. In engineering and manufacturing, mesh alignment is crucial for detecting deviations in machined parts, monitoring structural fatigue, and evaluating additive manufacturing precision [28]. The ability to measure intersecting volumes with high accuracy enables scientists and engineers to make data-driven decisions regarding design improvements, treatment efficacy, and material performance.

To achieve accurate volume assessment, advanced computational techniques are often employed. Voxel-based methods [29], signed distance functions [30], and finite element analysis [29] can enhance precision by capturing intricate surface geometries and subtle variations that might be overlooked with simpler approaches. Additionally, statistical and error estimation methods play a crucial role in validating results and ensuring that computed volume changes are significant rather than artifacts of measurement noise or misalignment [31]. Proper validation using control datasets, repeatability tests, and sensitivity analyses can further strengthen the reliability of volume assessments, making them suitable for high-stakes applications such as surgical planning, quality control, and forensic analysis.

Moreover, the integration of automation and machine learning in mesh alignment and volume assessment is becoming increasingly important. Automated feature extraction, deep learning-based registration algorithms [32], and AI-driven shape analysis [33] are enhancing the speed and accuracy of these processes, reducing manual intervention and improving reproducibility. The use of artificial intelligence in mesh processing can help identify patterns in volumetric changes that may not be immediately apparent through traditional methods, allowing for more detailed and nuanced interpretations of structural modifications. As computational power and algorithmic sophistication continue to evolve, mesh alignment and volume assessment techniques are expected to become even more precise and efficient, opening new possibilities for research and industrial applications.

Ultimately, the combination of precise mesh alignment and robust volumetric assessment techniques enables meaningful conclusions about structural changes to be drawn, facilitating advancements in a wide range of scientific and technological domains. Whether in medicine, engineering, or material sciences, the ability to accurately analyze intersecting volumes has far-reaching implications, influencing the development of better diagnostic tools, improved manufacturing processes, and more effective treatment strategies. In intraoral acquisitions, however, the scanned area does not always perfectly coincide between different sessions. Peripheral regions of the acquired meshes may vary due to operator-dependent scanning trajectories, limited access, or partial coverage of the anatomical field. These discrepancies, particularly at the margins of the scanned region, may introduce spurious volumetric components unrelated to the anatomical changes under investigation.

To ensure a consistent and anatomically meaningful localization of the volume of interest, we adopted a seed-point–based selection strategy. A seed point is manually defined on the reference mesh within the anatomical region intended for volumetric comparison. This point is required to lie inside the volumetric difference region generated between the two aligned meshes. During volumetric computation, only the connected volumetric component containing this seed point is retained, while any additional disconnected components—typically arising from peripheral mismatches or non-overlapping scan boundaries—are automatically excluded from the analysis.

## 3. Methods

### 3.1. 3D Analysis with Intraoral Scanners

**Figure 1:**
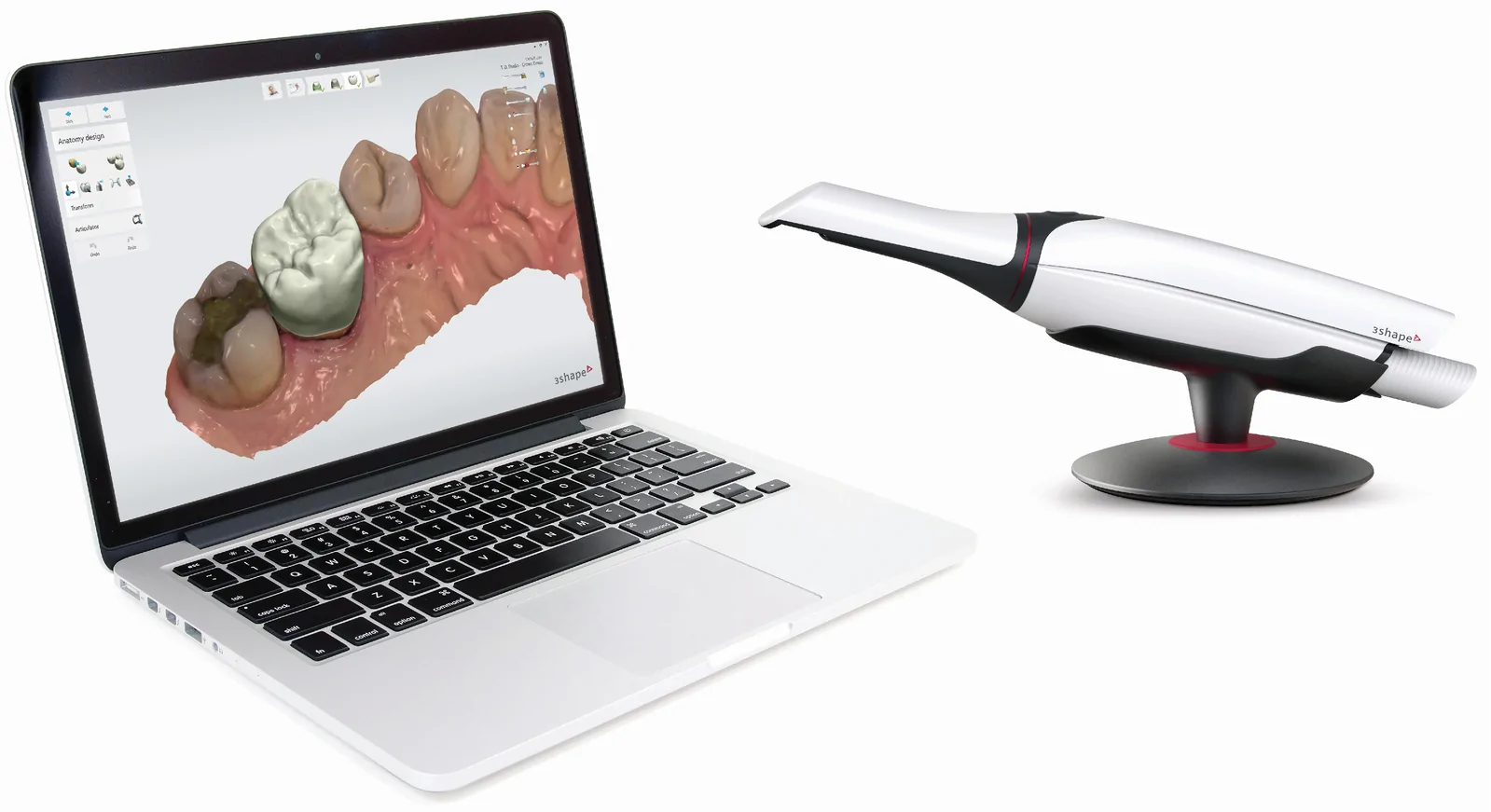
TRIOS 3 by 3Shape intraoral scanner used for the acquisitions.

**Figure 2:**
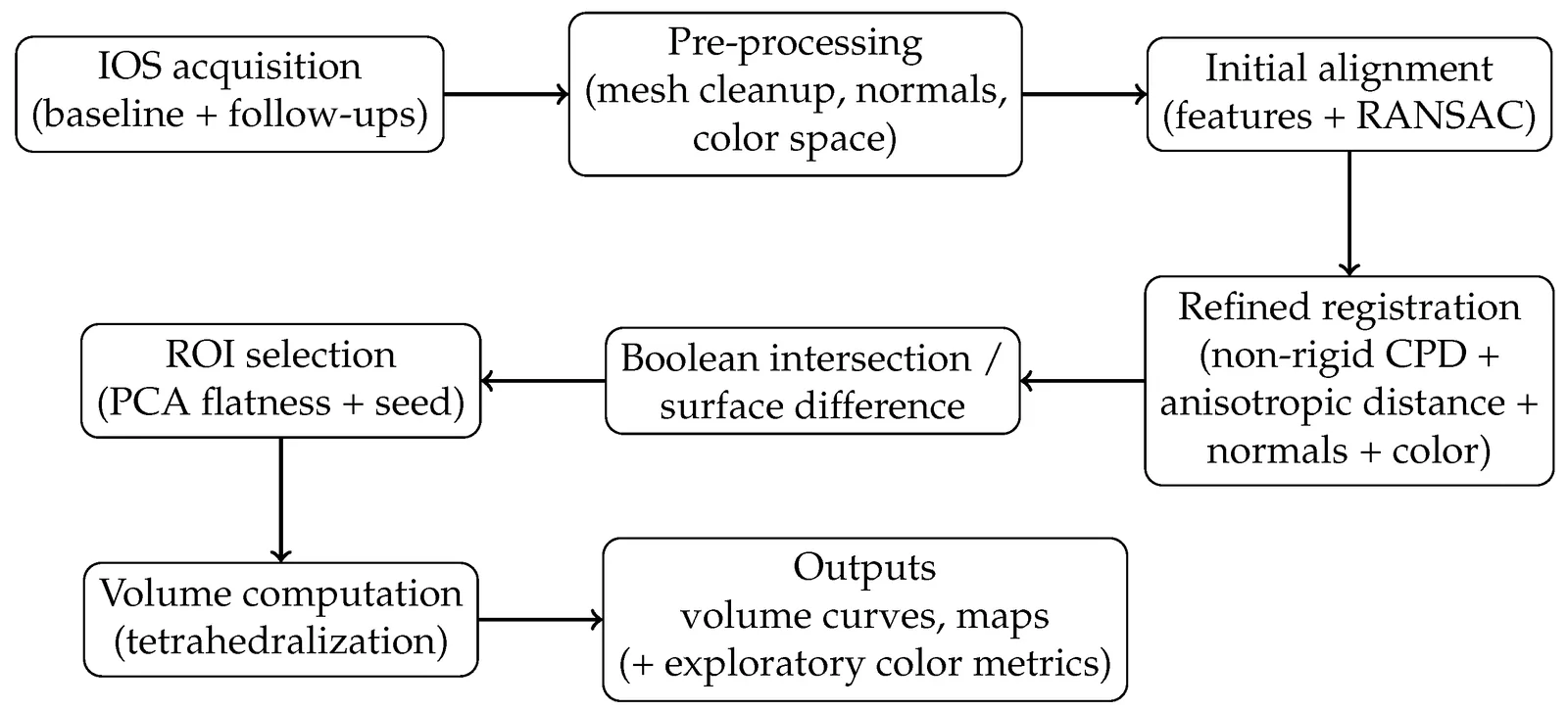
Overview of the proposed digital pipeline for longitudinal assessment of palatal donor site healing.

An intraoral 3D scanner (TRIOS 3, 3Shape; Figure 1) was used to acquire scans of the donor site at baseline, immediately after surgery, at 1 and 2 post-operative weeks, and at 1 and 3 months. The whole acquisition and processing pipeline is detailed in Figure 2.

### 3.2. Evaluation of System Accuracy

To further assess acquisition repeatability under clinically realistic conditions, an additional in vivo repeatability test was performed on healthy adult volunteers recruited among hospital staff. Five intraoral acquisitions were obtained, and each acquisition was repeated three consecutive times using the same TRIOS 3 scanner and the same acquisition protocol adopted for the clinical study. The repeated scans were processed using the same preprocessing, registration, and volumetric-analysis pipeline applied to the patient dataset.

Residual point-to-surface deviations were computed in anatomically stable regions, including enamel surfaces and non-operated palatal areas. The aggregate mean absolute deviation obtained from the repeated in vivo acquisitions was 0.21 ± 0.17 mm. This value is consistent with the sub-millimetric accuracy expected for the TRIOS 3 system and with previously published [22,34] accuracy and precision data for the TRIOS scanner series. Only the aggregate mean absolute deviation was retained in a standardized form for this supplementary repeatability assessment. Additional percentile-based residual metrics, such as RMS error, P90, and P95, were not available in a sufficiently standardized form and were therefore not reported. This limitation should be considered when interpreting small residual volumetric differences at later follow-up time points. Future prospective validation studies will include a predefined metrological protocol reporting mean absolute deviation, RMS error, P50, P90, P95, and volumetric sensitivity to ROI selection and registration parameters.

All intraoral scans were acquired using the TRIOS 3 scanner in normal acquisition mode, rather than high-resolution mode, in order to reduce acquisition time and improve patient comfort during intraoral procedures. Based on the repeated in vivo acquisitions and on the available literature, the expected instrumental and registration-related uncertainty of the workflow was considered to be in the sub-millimetric range. Therefore, residual volumetric differences at late follow-up time points were interpreted cautiously, especially when small in magnitude.

No additional validation was performed on synthetic materials, 3D-printed phantoms, or gravimetric models. Although such models are useful for controlled assessment of geometric trueness, they do not fully reproduce the optical and biomechanical properties of the real intraoral environment, including mucosal reflectance, moisture, saliva, enamel reflectivity, local curvature, and soft-tissue compliance. For this reason, a synthetic model could provide an estimate that is not fully representative of the clinical performance of the system. This limitation is acknowledged, and future studies will combine in vivo repeatability testing with phantom-based or gravimetric validation.

### 3.3. 3D Mesh Alignment

The goal of the alignment step is to bring two intraoral scans acquired at different time points (e.g., preoperative, immediate postoperative, and follow-up acquisitions) into a common coordinate system, so that subsequent surface/volume comparisons capture *true* anatomical changes. In our setting, registration is challenging for at least three reasons: (i) only a portion of the dental arch may be acquired at each time point; (ii) the dataset contains a mix of anatomically stable structures (primarily teeth) and deformable soft tissues; (iii) the palatal donor area undergoes genuine volumetric changes (wound cavity, edema, clot), which should *not* drive the alignment but should instead be treated as an outlier region during registration.

Given two consecutive scans, we denote by $M_{\mathrm{ref}}$ the reference mesh (typically baseline) and by $M_{\mathrm{mov}}$ the moving mesh (follow-up). Each mesh is represented by a set of vertices (point cloud) and the associated per-vertex attributes: surface normals and color. The rigid component of the alignment is represented by a homogeneous roto-translation matrix T∈SE(3).

#### 3.3.1. Pre-Processing

Meshes are first cleaned by removing isolated components and obvious scanning artefacts. Vertex normals are recomputed (or consistently re-oriented) from the mesh connectivity, which is essential both for normal-aware matching and for point-to-plane refinement. Color textures are converted to a perceptually meaningful color space (CIELAB) to enable the use of chromatic cues during refinement [35].

#### 3.3.2. Coarse Alignment: Global Rigid Initialization

A coarse estimate of T is required to avoid local minima in subsequent local/global refinement. While globally optimal or global registration methods can be used when the initial misalignment is large (e.g., Go-ICP [36], Fast Global Registration (FGR) [37], Super4PCS [38]), we adopt a feature-based initialization tailored to partially overlapping intraoral scans.

First, 3D keypoints are extracted on $M_{\mathrm{ref}}$ and $M_{\mathrm{mov}}$ based on local geometric saliency (e.g., curvature, normal variation) [39]. For each keypoint, we compute Fast Point Feature Histograms (FPFH) [40], which encode local angular geometry and have proven robust in the presence of moderate noise. Candidate correspondences are then established in feature space and a robust estimator (RANSAC [41]) is used to compute an initial rigid transformation $T_{0}$ by rejecting spurious matches.

#### 3.3.3. Coarse Alignment Quality Check with Hausdorff-Type Distances

To verify that $T_{0}$ yields a plausible overlap, we monitor a Hausdorff-type discrepancy between the transformed moving point set $T_{0}\;M_{\mathrm{mov}}$ and $M_{\mathrm{ref}}$. The directed Hausdorff distance between two point sets P and Q is defined as [42]:

(1) $$d_{H}^{\to }\left(P,Q\right)=\max_{p\in P}\;\min_{q\in Q}∥p-q∥.$$

The symmetric Hausdorff distance is obtained by taking the maximum of the two directed distances.

However, in our setting the two meshes are not expected to be fully overlapping, particularly in peripheral regions due to slightly different sampled areas. As a consequence, the full Hausdorff distance can be overly sensitive to outliers and non-corresponding boundary portions, where even a single mismatched point may dominate the metric. For this reason, we avoid relying on the classical (full) Hausdorff distance as a primary indicator of alignment quality.

Instead, we consider a robust variant based on high quantiles of the nearest-neighbor distance distribution (“modified” Hausdorff) [43], which mitigate the influence of outliers. In addition, distances can be optionally thresholded or evaluated after restricting the computation to an expected overlap region (e.g., by excluding peripheral areas or applying spatial clipping), further improving robustness to partial overlap.

In practice, these Hausdorff-type measures are used exclusively as coarse sanity checks—for instance, to detect gross misregistration—rather than as optimization objectives or absolute measures of geometric accuracy.

#### 3.3.4. Rigid Refinement with Normals and Robust Loss

Starting from $T_{0}$, we refine the rigid transform using a local method that exploits mesh structure and surface normals. Classical ICP [44] can be unstable when correspondences are contaminated by (i) partial overlap and (ii) true anatomical changes. To reduce sensitivity to these effects, we use point-to-plane refinement [45] together with a robust loss function.

Let $x_{i}\in M_{\mathrm{ref}}$ be a reference vertex with normal $n_{i}$ and let $y_{c(i)}$ be its closest-point correspondence on $T\;M_{\mathrm{mov}}$ under the current transform T. The point-to-plane residual is

(2) $$r_{i}\left(T\right)=n_{i}^{⊤}\left(x_{i}-T\;y_{c(i)}\right).$$

Rather than minimizing a pure least-squares objective $\sum_{i}r_{i}^{2}$, we minimize a robust M-estimation objective

(3) $$E_{\mathrm{rob}}\left(T\right)=\sum_{i}\rho \left(r_{i}\left(T\right)\right),$$

where ρ(·) is chosen to downweight large residuals associated with outlier regions (e.g., the donor site). Two standard choices are:

- Huber loss [46,47]:
  (4) $$\rho_{\delta }\left(r\right)=\left\{\begin{array}{cc} \frac{1}{2}r^{2}, & |r|\leq \delta ,\\ \delta \left(\left|r\right|-\frac{1}{2}\delta \right), & |r|>\delta , \end{array}\right.$$
  where δ sets the transition between quadratic (inlier) and linear (outlier) growth.
- Tukey biweight (bisquare) [47,48]:
  (5) $$\rho_{c}\left(r\right)=\left\{\begin{array}{cc} \frac{c^{2}}{6}\left[1-{\left(1-{\left(\frac{r}{c}\right)}^{2}\right)}^{3}\right], & |r|\leq c,\\ \frac{c^{2}}{6}, & |r|>c, \end{array}\right.$$
  which is redescending and effectively assigns zero influence to residuals larger than *c*.

This robust refinement is conceptually related to trimmed/overlap-based ICP variants developed for partially overlapping point sets [49]. For our case we adopted the Huber loss with δ=0.9mm.

#### 3.3.5. Refined Registration: Non-Rigid CPD with Mesh-, Normal-, Color- and Outlier-Awareness

After robust rigid refinement, residual discrepancies remain due to elastic deformations of soft tissues and to imperfect sampling. We therefore adopt the Coherent Point Drift (CPD) framework [50] as refined registration, extended to leverage (i) mesh connectivity, (ii) surface normals, (iii) chromatic information, and (iv) explicit outlier handling.

In CPD, the reference point set is $X_{N\times 3}={\left\{x_{i}\right\}}_{i=1}^{N}$ and the moving point set is $Y_{M\times 3}={\left\{y_{j}\right\}}_{j=1}^{M}$. Points $y_{j}$ are treated as centroids of a Gaussian Mixture Model (GMM) whose centroids are transformed to match X. An additional uniform component models noise/outliers.

##### Anisotropic (Mesh-Aware) Geometric Term

Standard CPD uses an isotropic Gaussian with variance $\sigma^{2}$. In our case, points follow a non-uniform sampling on a surface mesh, and deviations normal to the surface are more informative than tangential ones. To encode this, we replace the isotropic distance with a locally anisotropic Mahalanobis distance using a covariance matrix estimated from each mesh neighborhood [51]. For a correspondence (i,j), let $\Sigma_{i}$ and $\Sigma_{j}$ be the local covariance matrices estimated (e.g., from *k*-nearest mesh neighbors). We define a symmetric covariance

(6) $${\tilde{\Sigma }}_{ij}=\frac{1}{2}\left(\Sigma_{i}+\Sigma_{j}\right)+\epsilon \;I,$$

and the squared Mahalanobis residual

(7) $$r_{ij}^{2}={\left(x_{i}-{\hat{y}}_{j}\right)}^{⊤}\;{\tilde{\Sigma }}_{ij}^{-1}\;\left(x_{i}-{\hat{y}}_{j}\right),$$

where ${\hat{y}}_{j}$ denotes the transformed centroid.

##### Normal Consistency Term

To discourage matches across surfaces with incompatible orientations, we include a normal-consistency factor based on angular divergence:

(8) $$p_{\mathrm{norm}}\left(n_{x_{i}}|n_{{\hat{y}}_{j}}\right)=\exp\left(-\frac{\lambda }{2}\left[1-n_{x_{i}}^{⊤}n_{{\hat{y}}_{j}}\right]\right).$$

##### Chromatic Consistency Term

Textured IOS meshes provide a valuable cue to disambiguate stable enamel surfaces from mucosa, especially where geometric curvature is low. We incorporate color information via the $a^{*}$ channel of the CIELAB space [35], which captures green–red opponency:

(9) $$p_{\mathrm{color}}\left(a_{x_{i}}^{*}|a_{{\hat{y}}_{j}}^{*}\right)=\frac{1}{\sqrt{2\pi \sigma_{a}^{2}}}\exp\left(-\frac{{\left(a_{x_{i}}^{*}-a_{{\hat{y}}_{j}}^{*}\right)}^{2}}{2\sigma_{a}^{2}}\right).$$

##### Robust Outlier Suppression for True Volumetric Change Estimation

A key point for longitudinal palatal monitoring is that the donor site may undergo large, genuine shape changes. In registration terms, these points behave as structured outliers: they should be *ignored* for estimating the global transform, yet they must remain available for *post-alignment* volumetric analysis. To this end, we combine two robustification mechanisms: (i) the standard CPD “noise/outlier” component (uniform distribution) already reduces the influence of points that do not match any centroid well [50]; (ii) we additionally apply an M-estimation weight based on the geometric residual magnitude to downweight correspondences with large $r_{ij}$.

Concretely, in the EM iterations we compute a robust weight w(r) from the Huber function (Tukey could be used as an alternative as defined above) and use it to reweight the contribution of each correspondence in the M-step (an IRLS-style update) [47,48]. This has the practical effect of anchoring the alignment on stable structures (primarily teeth), while treating the donor site and swelling regions as outliers.

##### Combined Likelihood

The resulting (unnormalized) correspondence likelihood is proportional to

(10) $$p\left(x_{i}|{\hat{y}}_{j}\right)\;\propto \;\exp\left(-\frac{1}{2}r_{ij}^{2}\right)\;\cdot \;p_{\mathrm{norm}}\left(n_{x_{i}}|n_{{\hat{y}}_{j}}\right)\;\cdot \;p_{\mathrm{color}}\left(a_{x_{i}}^{*}|a_{{\hat{y}}_{j}}^{*}\right),$$

optionally multiplied by the robust weight $w\left(\sqrt{r_{ij}^{2}}\right)$ in the M-estimation step. Thanks to the differentiability of the above terms (and to the use of robust weights through reweighting rather than hard rejection), the optimization can still be performed efficiently using the Expectation–Maximization scheme underlying CPD [50].

### 3.4. Volumetric Analysis of the Healing Steps

The boolean procedure can produce several closed components. Some of them are associated with the actual surgical donor site, whereas others may arise from residual registration errors, acquisition noise, partial scan overlap, peripheral mismatches, small anatomical variations of the oral mucosa, or non-surgical changes occurring between acquisitions. In longitudinal intraoral datasets, a fully automatic selection of the donor-site component may therefore be ambiguous. For example, differences in scan coverage or changes involving dental surfaces, such as restored, partially missing, or newly visible tooth regions, may generate additional closed components unrelated to palatal wound healing. These components may occasionally show geometric properties similar to those of the donor-site defect and cannot always be reliably excluded using a single automatic descriptor.

For this reason, the region of interest was identified using a semi-automatic seed-guided component-selection strategy. The ROI was not manually contoured. Instead, a single seed point was manually selected by a trained operator on a rendered view of the registered meshes, within the clinically identified palatal donor-site region. The same operator performed this step for all datasets to maintain procedural consistency. The seed point was used only to identify the connected closed component corresponding to the donor site; it did not define the boundary of the ROI and did not modify the geometry of the selected mesh.

The two-dimensional seed selected in the rendered view was converted into a three-dimensional selection by ray casting. Let

$$u_{s}=\left(u_{s},v_{s}\right)$$

be the image coordinates of the selected seed point. Given the virtual camera center of the rendering window, denoted by $o_{c}$, and the viewing direction associated with the selected image point, denoted by $d(u_{s})$, the corresponding back-projection ray was defined as

$$l_{s}\left(\tau \right)=o_{c}+\tau \;d\left(u_{s}\right),\;\tau >0.$$

The first intersection of this ray with the visible registered surface provided the three-dimensional seed location. The same ray was then tested against the closed volumetric components obtained from the surface-intersection procedure. The candidate set of seed-compatible components was defined as

$$K_{s}=\left\{k\;:\;l_{s}\cap \partial \Omega_{k}\neq \emptyset \right\},$$

where $\Omega_{k}$ denotes the *k*-th closed volumetric component and $\partial \Omega_{k}$ its triangular boundary surface. Components not intersected by the seed ray were excluded from the donor-site volume computation.

Principal component analysis (PCA) was then used as a geometric consistency criterion for the seed-compatible components [52]. The residual defect generated by palatal soft-tissue harvesting is expected to be a relatively flat volumetric region, with one spatial dimension substantially smaller than the other two. For each candidate component, the covariance matrix of its vertices was computed and diagonalized. Let

$$\lambda_{1}\geq \lambda_{2}\geq \lambda_{3}$$

be the eigenvalues associated with the three principal axes. The flatness score was defined as

$$\rho =\frac{\lambda_{3}}{\lambda_{1}}.$$

A small value of ρ indicates that the vertices are mainly distributed along two dominant directions, while showing limited variation along the third one. Therefore, when multiple seed-compatible components were present, the final donor-site component was selected as

$$k^{⋆}=\arg\min_{k\in K_{s}}\rho_{k}.$$

When only one seed-compatible component was identified, PCA was used as a consistency check to verify that its geometry was compatible with the expected flat morphology of the donor-site defect.

This procedure combines anatomical guidance and automatic geometric filtering. The manually selected seed point provides a clinically meaningful localization of the donor-site region, whereas the PCA-based flatness score reduces the risk of retaining components caused by peripheral scan mismatch or non-surgical surface changes. Importantly, the seed point does not determine the ROI boundary: the final boundary is entirely defined by the closed component generated by the surface-intersection procedure. Consequently, small variations in the exact seed-point position do not affect the computed volume as long as the seed ray selects the same connected component.

Although this classifier is intentionally simple, it was sufficient for the cases considered in this pilot feasibility study. More robust classifiers could also include additional geometric descriptors, such as surface area, enclosed volume, surface-to-volume ratio, bounding-box dimensions, spatial proximity to the clinically identified donor site, or local curvature-based features.

After rigid alignment of all the meshes acquired at the different healing checkpoints, the volume variation between the pre-surgical anatomy and each post-surgical scan was estimated. Let $M_{0}$ denote the pre-surgical mesh and $M_{t}$ the mesh acquired at a given healing checkpoint. The target volume is the space enclosed between the two surfaces in the donor region, i.e., the residual defect volume at time *t*.

**Figure 3:**
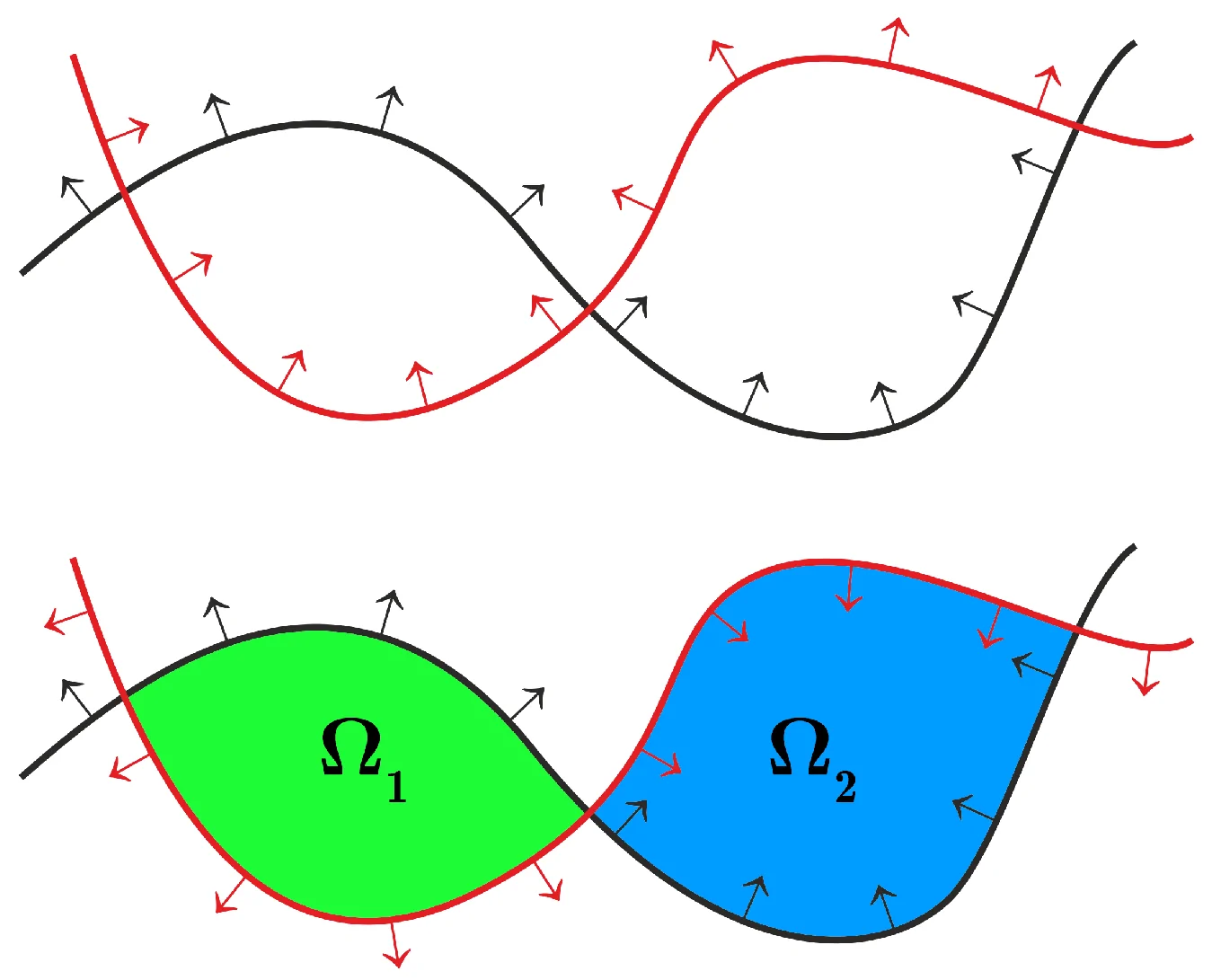
Schematic representation of the signed volumetric difference between two aligned meshes. The pre-surgical reference mesh $M_{0}$ is shown in red, whereas the follow-up mesh $M_{t}$ is shown in black. Arrows represent the surface normals. After reversing the normal orientation of $M_{t}$, the intersection between the two surfaces defines closed volumetric regions. The region $\Omega_{1}$ corresponds to a local tissue volume increase: its boundary is oriented with outward-pointing normals, resulting in a positive signed volume according to the divergence theorem. Conversely, the region $\Omega_{2}$ represents a local volume reduction, for which the boundary normals are inward-oriented with respect to the same convention, yielding a negative signed volume.

Since intraoral scans provide open surface meshes, the definition of a closed volumetric region depends on the consistency of the surface normals. In the adopted convention, the normals of the checkpoint mesh $M_{t}$ were flipped inward with respect to the acquired surface, whereas the normals of the pre-surgical mesh $M_{0}$ were kept oriented outward. This orientation makes the two surfaces locally delimit the enclosed cavity between the original anatomy and the healing surface (see Figure 3), Without this normal configuration, the boolean operation may lead to ambiguous or incorrectly oriented components, preventing the construction of a well-defined closed volume [53].

The volume of the selected closed mesh was computed using the divergence theorem. For a closed surface ∂V with outward unit normal $\hat{n}$, choosing the vector field

$$F\left(x\right)=\frac{1}{3}x$$

gives

∇·F=1.

Therefore, the volume enclosed by the surface can be written as

$$V=\frac{1}{3}{\;\int \int \;}_{\partial V}x\cdot \hat{n}\;dS.$$

Since the surface is represented by a triangular mesh, the surface integral can be evaluated as a sum of signed tetrahedral volumes. For a closed mesh composed of *N* oriented triangular faces, with vertices $v_{i1}$, $v_{i2}$, and $v_{i3}$, the enclosed volume is

$$V=\frac{1}{6}\sum_{i=1}^{N}v_{i1}\cdot \left(v_{i2}\times v_{i3}\right).$$

Each term represents the signed volume of the tetrahedron formed by the triangle and the origin. For a consistently oriented closed mesh, the algebraic sum cancels the external contributions and returns the volume enclosed by the surface. The absolute value guarantees a positive result in case the global orientation of the final mesh is reversed.

All the steps described above, including mesh cutting, triangular remeshing, component extraction, PCA-based filtering, and volume computation, were implemented in Python 3.14 using the PyVista library(vers. 0.48) [54].

## 4. Results

This pilot feasibility study included six patients in good general health who underwent palatal soft-tissue harvesting. For each patient, the palatal donor site was monitored through a longitudinal sequence of intraoral 3D scans acquired at six time points: pre-operative baseline ($t_{0}$), immediate post-operative acquisition ($t_{1}$), 1 week ($t_{2}$), 2 weeks ($t_{3}$), 1 month ($t_{4}$), and 3 months ($t_{5}$). All follow-up meshes were registered to the corresponding baseline scan, allowing local morphological changes of the donor area to be evaluated in a common reference system.

**Figure 4:**
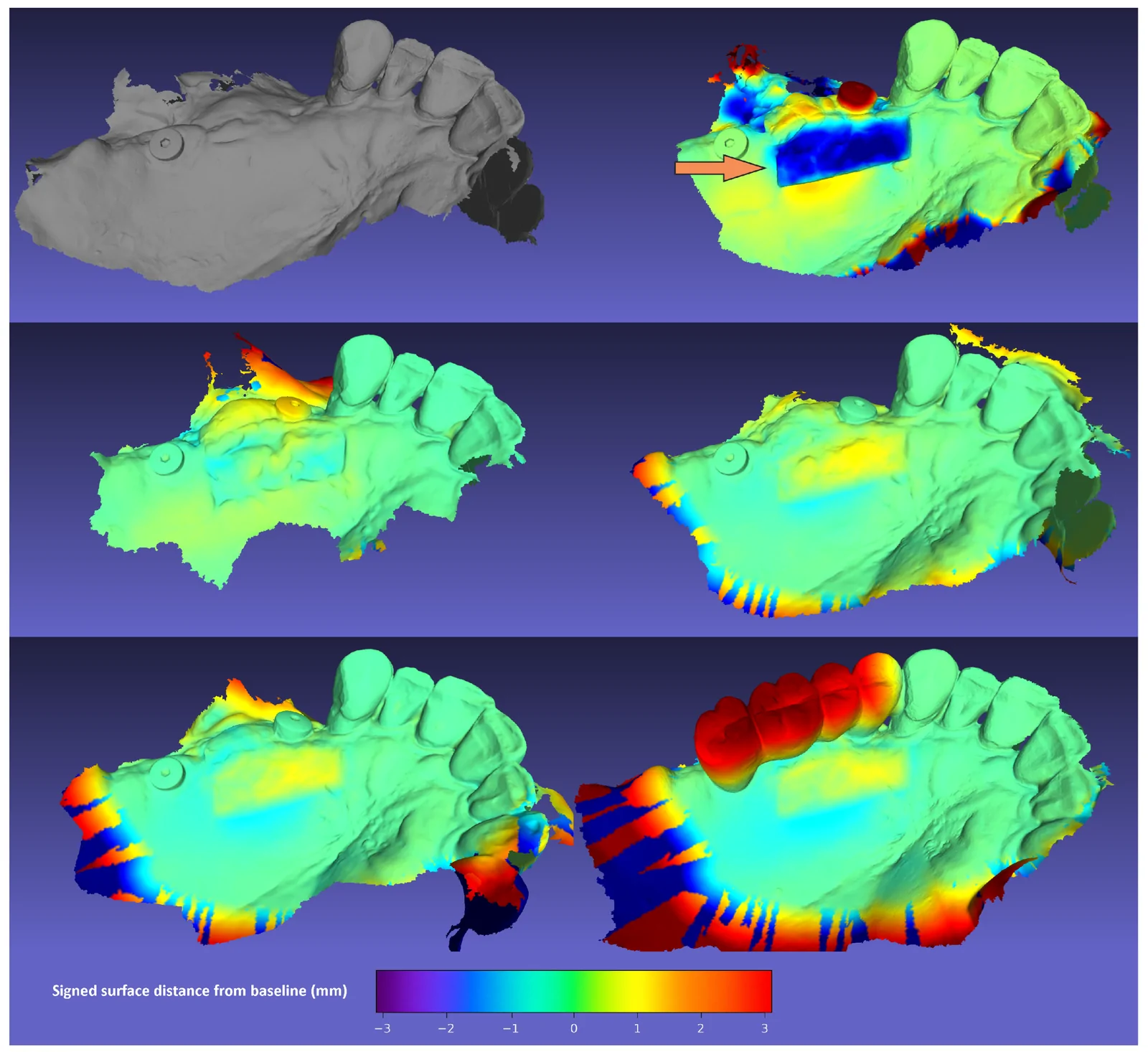
Representative longitudinal surface-deviation maps of the palatal donor site for Patient 1. The six panels are ordered from the top-left to the bottom-right and show the temporal evolution from the pre-operative baseline scan ($t_{0}$) to the 3-month follow-up ($t_{5}$). The $t_{0}$ panel is shown as the reference surface, whereas the follow-up panels are color-coded according to the signed surface distance from the baseline scan, expressed in millimeters. Negative values indicate inward displacement of the follow-up surface with respect to baseline, corresponding to local tissue deficit, whereas positive values indicate outward displacement, corresponding to apparent local tissue increase or swelling. The color scale was clipped to the range −3 to +3 mm for visualization. The palatal donor-site region is marked by an arrow to facilitate visual identification; this marking was used only for visualization and did not define the computational ROI used for volume calculation.

**Figure 5:**
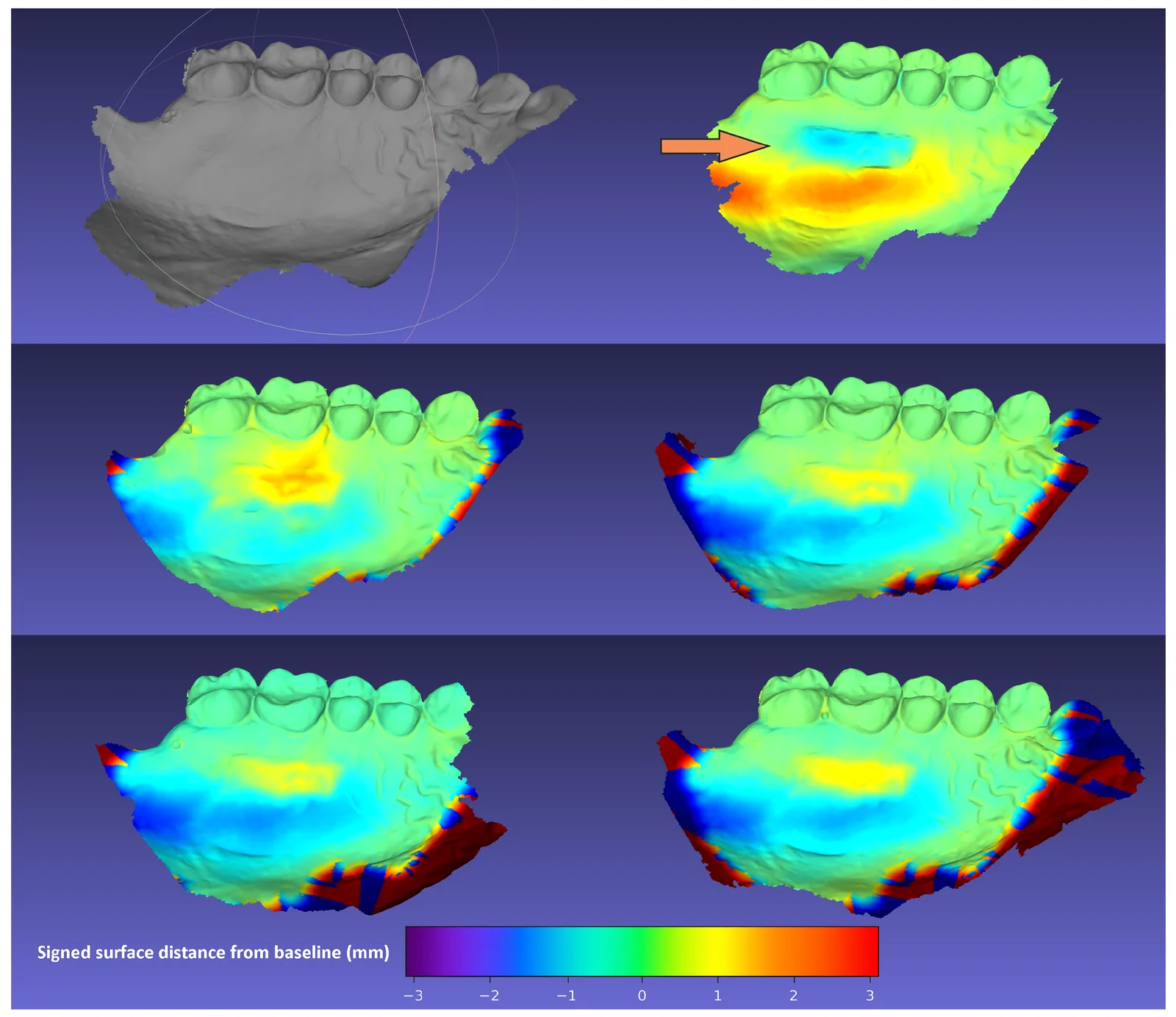
Representative longitudinal surface-deviation maps of the palatal donor site for Patient 2. The six panels show the registered intraoral scans at $t_{0}$, $t_{1}$, $t_{2}$, $t_{3}$, $t_{4}$, and $t_{5}$. The color map represents the signed surface distance from the pre-operative baseline scan, expressed in millimeters. Negative values indicate inward displacement of the follow-up surface with respect to baseline, corresponding to local tissue deficit, whereas positive values indicate outward displacement, corresponding to apparent local tissue increase or swelling. The color scale was clipped to the range −3 to +3 mm for visualization. The donor-site region is marked by an arrow to make the clinically relevant area more evident. This visual marking was not used to define the computational ROI, which was selected using the seed-guided component-selection procedure described in the Methods.

Figure 4 and Figure 5 show two representative examples of the temporal evolution of the palatal surface after soft-tissue harvesting. Each figure is composed of six subfigures, ordered from the top-left to the bottom-right, corresponding to the successive acquisition time points from $t_{0}$ to $t_{5}$. The same anatomical region is therefore visualized repeatedly over time, making it possible to qualitatively assess the spatial distribution of local tissue gain and loss during the healing process.

In the early post-operative phase, the donor site generally showed the largest deviation from the baseline geometry, mainly reflecting the tissue volume removed during graft harvesting. However, in some cases the signed volume measured immediately after surgery was higher than the pre-operative baseline. This apparent volume gain should not be interpreted as true tissue regeneration, which is not expected at such an early stage, but rather as the effect of the acute post-operative response. Surgical trauma may induce local vasodilation and increased vascular permeability, leading to inflammatory edema and soft-tissue swelling; additionally, blood clot formation or incomplete hemostasis may transiently increase the apparent volume of the donor area. As healing progresses, these early post-operative effects tend to resolve, and the measured surface morphology progressively stabilizes toward the baseline configuration.

In volumetric terms, the largest differences were observed between the pre-operative and immediate post-operative scans. Five patients showed a negative signed volumetric variation consistent with the amount of tissue harvested and the thickness of the graft. Conversely, one patient exhibited a positive immediate post-operative volumetric change of approximately $13\;{\mathrm{mm}}^{3}$. This finding is likely attributable to the acute inflammatory response induced by surgery, including local vasodilation, edema, soft-tissue swelling, and possible clot formation, rather than to newly formed gingival tissue.

Patient 3 represented a specific early post-operative exception. Although this patient did not show a positive signed volume immediately after surgery, an isolated positive peak of $+81\;{\mathrm{mm}}^{3}$ was observed at $t_{2}$, corresponding to the 1-week follow-up. The harvested area in this patient was particularly deep, and clinical examination at 1 week showed marked inflammation and visible edema of the donor site. Therefore, the $t_{2}$ peak was interpreted as an apparent volume increase related to persistent inflammatory swelling and edema, possibly associated with clot/scab formation, rather than as true tissue regeneration. The subsequent return to negative values at $t_{3}$ supported the transient nature of this finding.

**Figure 6:**
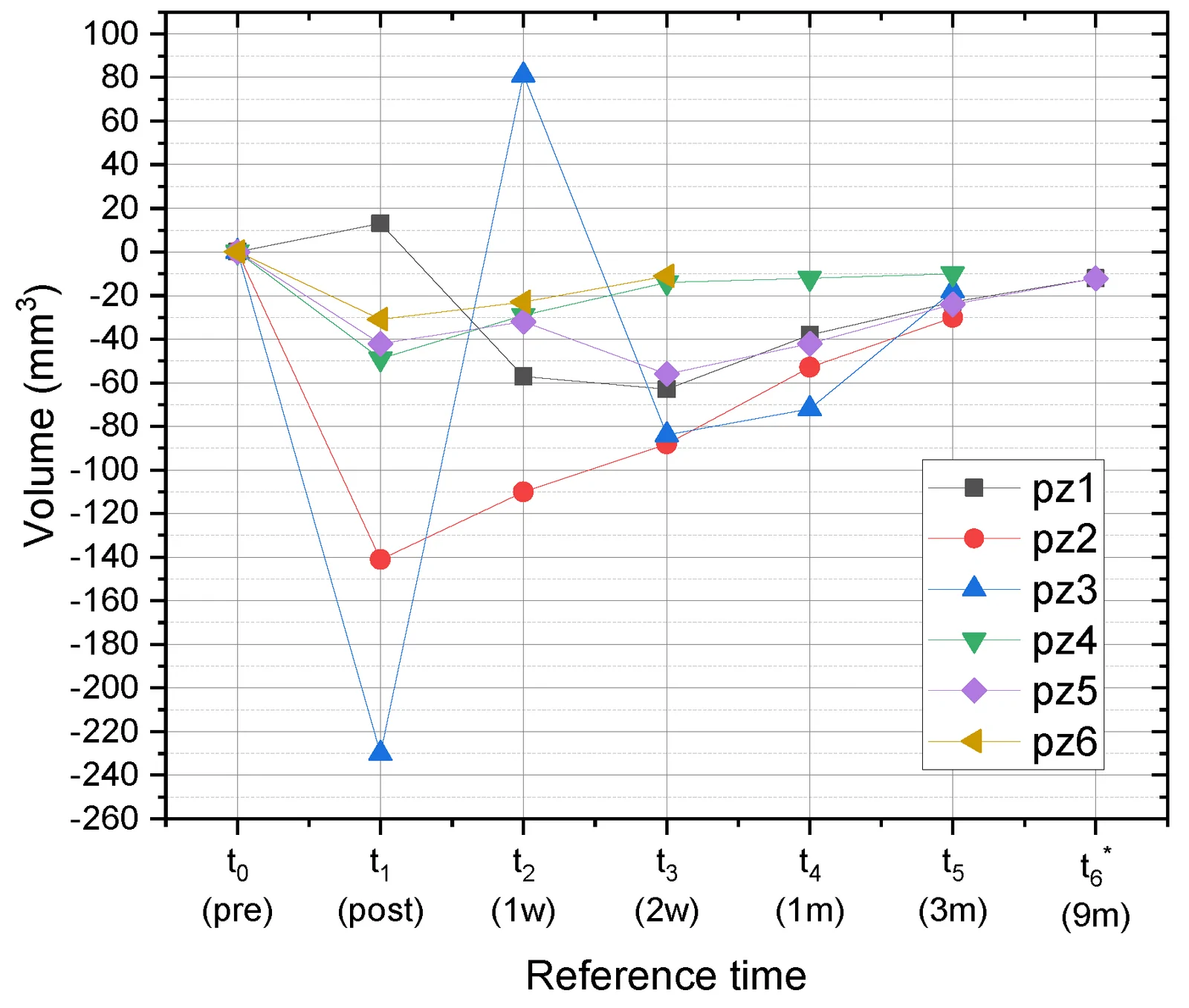
Signed volumetric changes measured at the palatal donor site for the six patients during the follow-up period. Negative values indicate a volumetric deficit with respect to the baseline scan, whereas positive values indicate a local apparent volume increase. Early positive values should be interpreted as transient post-operative effects, potentially associated with vasodilation, inflammatory edema, tissue swelling, blood clot formation, or incomplete hemostasis, rather than as true tissue regeneration. The additional late follow-up point $t_{6}^{*}$ is reported only for the two patients for whom an extended follow-up scan was available; it is shown solely to illustrate the longer-term trend at 9 months of volumetric recovery and was not included in the group summary statistics.

The signed volumetric changes measured for all six patients are summarized in Figure 6. Overall, the palatal donor site showed a progressive tendency to recover its initial morphology over time. Although patient-specific variability was observed, the absolute volumetric discrepancy generally decreased during the follow-up period, with the donor site approaching the baseline configuration at the 3-month evaluation. This quantitative trend confirms that the proposed workflow is able to provide a detailed and objective description of the dynamic healing process, complementing the visual inspection of the registered 3D models.

### 4.1. Clinical Assessment of Wound Healing

Clinical healing of the palatal donor site was assessed by evaluating the degree of re-epithelialization at each follow-up time point. The distribution of partial and complete re-epithelialization is summarized in Table 2.

At $t_{2}$, corresponding to the 1-week follow-up, four patients showed partial re-epithelialization of the palatal donor site, whereas no patient showed complete epithelial closure. At $t_{3}$, corresponding to the 2-week follow-up, five patients showed partial re-epithelialization, while one patient had already achieved complete re-epithelialization. At $t_{4}$, corresponding to the 1-month follow-up, five patients were clinically evaluated: two patients showed complete re-epithelialization and three patients still showed partial re-epithelialization. At $t_{5}$, corresponding to the 3-month follow-up, complete re-epithelialization was observed in all clinically evaluated patients.

These clinical observations indicate a gradual progression of epithelial healing over time. However, clinical epithelial closure and three-dimensional volumetric recovery should be considered complementary but not equivalent aspects of palatal donor-site healing.

At $t_{5}$, corresponding to the 3-month follow-up, complete re-epithelialization was observed in all five clinically evaluated patients. For Patient 1 and Patient 5, an additional 9-month follow-up scan was available and is reported in Figure 6 as $t_{6}^{*}$. Both patients showed a residual signed volumetric deficit of −12 mm^3^, suggesting continuation of the recovery trend. However, because this late time point was available only for two patients, it was considered exploratory and was not included in the group summary statistics.

### 4.2. Three-Dimensional Volumetric Analysis of the Donor Site

The analysis of the different healing stages was performed by evaluating the signed soft-tissue volume change at each follow-up with respect to the corresponding baseline scan. The individual signed volumetric values for all patients are reported in Table 3, together with mean, standard deviation, and range at each time point.

Immediately after surgery ($t_{1}$), the mean signed volume change was −80.0 ± 89.1 mm^3^ compared with baseline, with values ranging from −230 to 13 mm^3^. At the 1-week follow-up ($t_{2}$), the mean signed change was −28.3 ± 62.4 mm^3^. The large variability at this time point was mainly related to Patient 3, who showed a positive value of 81 mm^3^ in association with clinically evident inflammation and edema of the donor site. At 2 weeks ($t_{3}$), the mean signed volume change was −52.7 ± 33.4 mm^3^.

For five patients, a 1-month follow-up ($t_{4}$) was available, showing a mean residual signed volumetric deficit of −43.4 ± 21.9 mm^3^. At the 3-month follow-up ($t_{5}$), five patients were available and the mean residual signed volumetric deficit decreased to −21.0 ± 7.5 mm^3^. The additional late follow-up point $t_{6}^{*}$ is reported only for the two patients for whom an extended follow-up scan was available at 9-month follow-up; Missing values indicate unavailable follow-up scans and were not included in the calculation of the summary statistics.

## 5. Discussion

To date, the techniques used in clinical practice and described in literature for correcting mucogingival tissues to create adequate soft tissue volumes or correct periodontal phenotype are numerous. Connective tissue graft (CTG) is one of the most common types of grafts for increased keratinized tissue, both around natural teeth and implants [55]. Studies in literature have shown that, unlike sites treated without any tissue augmentation procedure, areas treated with CTG result in a greater thickness of soft tissues, although there is a similar marginal bone loss (MBL) [56]. There are also collagen matrices which, in some cases, can replace the connective tissue graft, eliminating a second surgical site and thus causing less discomfort to the patient. However, a recent meta-analysis work, conducted by Cairo has shown that sites treated with CTG show a higher amount of keratinized tissue, lower inflammation and lower MBL [57]. In some cases, a second CTG may be needed to correct soft tissue defects at the same site or in different areas. Soileau and Brannon stated that it is possible to perform a re-harvesting of connective tissue not earlier than 9 weeks after the first surgery, otherwise the autogenous graft quality may be impaired [58]. Tavelli et al. indicate that a longer healing time may be needed for the palatal donor site to reach the preharvesting volume and, therefore, it is not recommended to perform a second CTG from the same donor area before 3 months of healing [3]. After the surgery, the wound on the palate is healed by second intention and some studies show that the cell migration proceeds from the most anterior to the most posterior, leaving the central area to heal last [59]. The re-epithelialization of the palatal donor site was gradual, with partial healing for all patients within the first month and complete epithelial closure only by the third month. This pattern reflects the normal healing process of the oral mucosa, which takes weeks or months to complete [60]. The three-dimensional volumetric analysis of the palatal donor site is a key aspect to evaluate the healing process of the palatal mucosa because it allows objective quantification of the reduction of the volume of the surgical site, giving precise indications on the course of tissue healing over time. The edema and bleeding were found to be most common postoperative complications: the occurrence of these events has complicated the volumetric analysis of the affected areas, especially in the immediate post-operative period and at 7-day follow-up. Positive signed volumes were observed only in isolated early post-operative measurements. One patient showed a small positive value immediately after surgery, whereas Patient 3 showed a marked positive peak at the 1-week follow-up, associated with clinically evident inflammation and edema. Although this temporarily resulted in an increase in tissue volume, these findings reflect the normal postoperative variability found in routine clinical practice. In this pilot cohort, the palatal donor site showed a progressive tendency toward volumetric recovery during follow-up. At 3 months, complete clinical re-epithelialization was observed, although a small residual mean volumetric deficit was still measurable. This finding suggests that epithelial closure and complete volumetric recovery may not occur simultaneously. Therefore, the persistence of a residual volumetric deficit should be considered when evaluating the possibility of re-harvesting from the same palatal donor area. However, because of the small sample size and the limited 3-month follow-up, the present data cannot define an evidence-based minimum healing interval for a second graft harvest. Decisions regarding re-harvesting should remain based on clinical judgment, individual donor-site assessment, and patient-specific healing conditions. The data obtained from this study are in line with what was reported by Tavelli, which indicates that a complete healing and maturation of the palatal mucosa at the donor site occurs only after 6–12 months [3]. The main limitation of the present study is the small sample size. Only six patients were included; therefore, the results should be interpreted as preliminary and hypothesis-generating. The study was designed as a pilot feasibility investigation to evaluate whether longitudinal intraoral scanning combined with three-dimensional registration and signed volumetric analysis could objectively describe palatal donor-site healing. It was not powered to establish definitive clinical thresholds, to quantify inter-individual healing variability, or to determine the optimal timing for a second graft harvest from the same donor area. In addition, the follow-up was limited to 3 months, whereas complete maturation of palatal soft tissues may require longer observation periods. Future studies should include larger prospective cohorts, standardized surgical and acquisition protocols, and longer follow-up intervals to determine whether residual volumetric deficits can be used to guide clinical decisions regarding re-harvesting.

## 6. Conclusions

This pilot feasibility study presented a digital three-dimensional workflow for monitoring volumetric changes in the palatal donor site after soft-tissue harvesting. By combining longitudinal intraoral scans, robust mesh registration, region-of-interest selection, and signed volume computation, the proposed approach enabled non-invasive quantification of local morphological changes during wound healing in a small cohort of six patients.

The results suggest that complete clinical re-epithelialization and volumetric recovery should be considered complementary but not equivalent aspects of donor-site healing. In particular, a residual volumetric deficit may persist even after visual epithelial closure. However, because of the limited sample size and the 3-month follow-up period, these findings should be considered preliminary. The workflow appears feasible for longitudinal monitoring, but larger prospective studies with longer follow-up are required before definitive clinical recommendations can be made, especially regarding the timing of a possible second graft harvest from the same palatal donor area.

## Figures and Tables

**Table 2:** Clinical re-epithelialization of the palatal donor site during follow-up.

Time Point	Available Patients	No Evident/Initial	Partial	Complete
$t_{2}$, 1 week	6	2	4	0
$t_{3}$, 2 weeks	6	0	5	1
$t_{4}$, 1 month	5	0	3	2
$t_{5}$, 3 months	5	0	0	5

Clinical categories refer to visual assessment of the palatal donor site. “No evident/initial” indicates that complete or clearly partial re-epithelialization was not clinically appreciable at that time point. Missing clinical evaluations were not included in the corresponding row.

**Table 3:** Individual signed volumetric changes of the palatal donor site at each post-operative time point. Values are expressed in mm^3^ and refer to the difference with respect to the pre-operative baseline scan. Negative values indicate a residual volumetric deficit, whereas positive values indicate an apparent local volume increase.

Time Point	Pt 1	Pt 2	Pt 3	Pt 4	Pt 5	Pt 6	*n*	Mean	SD	Range
$t_{1}$, post-op.	13.2	−141.3	−229.8	−49.2	−42.0	−31.1	6	−80.0	89.1	−230 to 13
$t_{2}$, 1 week	−56.9	−109.9	81.4	−29.2	−32.3	−22.9	6	−28.3	62.4	−110 to 81
$t_{3}$, 2 weeks	−63.0	−87.7	−84.2	−13.6	−56.1	−10.8	6	−52.7	33.4	−88 to −11
$t_{4}$, 1 month	−38.1	−53.0	−71.8	−12.0	−41.8	–	5	−43.4	21.9	−72 to −12
$t_{5}$, 3 months	−23.0	−30.0	−18.2	−10.1	−24.3	–	5	−21.0	7.5	−30 to −10

SD was computed using the available patients at each time point. Missing values indicate unavailable follow-up scans and were not included in the summary statistics.

## Data Availability

Data can be made available upon reasonable request to the corresponding author.
